# ASSESSMENT OF NUTRITIONAL STATUS AND FREQUENCY OF COMPLICATIONS ASSOCIATED TO FEEDING IN PATIENTS WITH SPASTIC QUADRIPLEGIC CEREBRAL PALSY

**DOI:** 10.1590/1984-0462/2020/38/2018410

**Published:** 2020-05-11

**Authors:** Kamilla Tavares de Sousa, Gabrielle Bemfica Ferreira, Amanda Torido Santos, Quintiliano Siqueira Schroden Nomelini, Luciana Oliveira de Almeida Minussi, Érica Rodrigues Mariano de Almeida Rezende, Isabella Lopes Nonato

**Affiliations:** a Universidade Federal de Uberlândia, Uberlândia, MG, Brazil.; b Centro Universitário do Triângulo, Uberlândia, MG, Brazil.

**Keywords:** Cerebral palsy, Nutritional status, Feeding, Diet, Enteral nutrition, Paralisia cerebral, Estado nutricional, Alimentação, Dieta, Nutrição enteral

## Abstract

**Objective:**

To correlate the nutritional status with variables associated to the type of diet and feeding route of children and adolescents with spastic quadriplegic cerebral palsy (CP).

**Methods:**

This cross-sectional study included 28 patients aged ≤13 years old who presented a diagnosis of spastic quadriplegic CP and were followed by the nutrition team of the Outpatient Clinic for Special Patients of Hospital de Clínicas de Uberlândia - Universidade Federal de Uberlândia (HC-UFU), between July/2016 and January/2017. Consent forms were signed by the legal guardians. The nutritional status was evaluated and data on dietary complications food route and type of diet were collected. For the description of data, average and median values were used. Correlation was tested with Spearman’s index. Significance was set at p<0.05.

**Results:**

75% of patients used alternative feeding routes (nasoenteral, catheter or gastrostomy), 57% were eutrophic. The most frequent complications were oropharyngeal dysphagia, reflux and intestinal constipation. No correlation was found between the occurrence of complications and the nutritional status. There was a positive correlation between the diet received and the patient’s nutritional status (0.48; p=0.01), i.e. individuals with adequate caloric and macronutrients intake had a better nutritional status.

**Conclusions:**

The results reinforce the need for continued nutritional guidance for the children’s parents/caregivers, as well as the choice of an adequate rout of feeding to each child by the multi-professional team, in order to contribute to improved nutritional status and adequate dietary intake.

## RESUMO

**Objetivo:**

Correlacionar o estado nutricional com variáveis associadas ao tipo de dieta e via de alimentação de crianças e adolescentes com paralisia cerebral (PC) tetraparética espástica.

**Métodos:**

Estudo de corte transversal com 28 pacientes com idade ≤13 anos, atendidos pela equipe de nutrição do Ambulatório de Pacientes Especiais do Hospital de Clínicas de Uberlândia da Universidade Federal de Uberlândia (HC-UFU), entre julho de 2016 e janeiro de 2017, que apresentavam diagnóstico de PC tetraparética espástica. Os pacientes incluídos tiveram o termo de consentimento assinado pelo responsável legal. Avaliou-se o estado nutricional e coletaram-se dados sobre complicações alimentares, via de alimentação e tipo de dieta. Para descrição desses dados, foram utilizadas média e mediana; para análises de correlação, correlação de Spearman, sendo significante p<0,05.

**Resultados:**

Neste estudo, 75% dos pacientes utilizavam via alternativa para alimentação (sonda nasoenteral ou gastrostomia) e 57% eram eutróficos. As complicações mais frequentes foram disfagia orofaríngea, refluxo e obstipação intestinal. Não houve correlação entre ocorrência de complicações e estado nutricional. Observou-se correlação positiva entre dieta recebida e estado nutricional dos pacientes (0,48; p=0,01), sendo que indivíduos com adequada ingestão calórica e de macronutrientes apresentaram melhor classificação do estado nutricional.

**Conclusões:**

Os resultados obtidos reforçam a necessidade de orientações nutricionais contínuas para os pais/cuidadores das crianças do estudo, bem como a escolha de uma via de alimentação adequada a cada uma delas pela equipe multiprofissional, a fim de contribuir para a melhora do estado nutricional e da ingestão alimentar.

## INTRODUCTION

Cerebral palsy (CP), also known as chronic non-progressive encephalopathy, is characterized by a set of brain disorders caused by non-progressive and irreversible injuries, mainly resulting from neonatal asphyxia, congenital infections and trauma. It occurs in the developing brain (up to three years old) and can contribute to limitations in the individual’s functional profile, in addition to significantly compromising the neurological system.^1^ ^,^ ^2^

According to its dominant clinical characteristic, CP can be classified as spastic, dyskinetic or ataxic.^3^ The spastic type is the most reported in the literature and is characterized by increased muscle tone. Spastic CP is caused by injury to the pyramidal system, classified according to its anatomical distribution into unilateral (monoplegia or hemiplegia) and bilateral (diplegia, triplegia or quadriplegia).^4^ Individuals affected by spastic CP and bilateral anatomical distribution with tetraplegia, that is, spastic quadriplegia, present global alteration of muscular tone and decrease in spontaneous motor and articular mobility that propitiate the appearance of several corporal deformities.^2^

Children and adolescents diagnosed with spastic quadriplegic CP have several risk factors and dietary and health complications, which can hinder or even prevent proper nutrition.^5^ Among the complications associated to food, we can mention oropharyngeal dysphagia, aspiration pneumonia, intestinal constipation and gastroesophageal reflux disease (GERD).^6^ The presence of one or more complications related to eating can lead to serious nutritional disorders and, in many cases, the need for alternative ways of eating. The use of these alternative routes is indicated to prevent the development of malnutrition,^7^ with hypoactivity and increased rates of respiratory infections, with a consequent increase in morbidity.^8^

In some studies, it has been shown that children with CP may experience malnutrition.^5^ Malnutrition in patients with CP is often associated to low energy intake. Often, this low energy intake is related to the prescription of qualitatively adequate diets, but to inadequate volumes and/or route of administration.^7^ The choice of route of administration of diets depends on the clinical conditions related to swallowing, the integrity of the gastrointestinal tract and the general condition of the patient.^8^ Thus, the choice of the diet of patients with CP, as well as the type and volume of diet prescribed and administered, is fundamental for maintaining or recovering the nutritional status of these patients. Therefore, the presence of a multidisciplinary team is necessary to carry out the treatment and monitoring of children and adolescents with CP.^8^

Although great advances in the interdisciplinary approach to the health care of children and adolescents with CP have been already accomplished, studies on complications resulting from food and its impact on the nutritional status of these individuals are still scarce. Due to the need to promote an improvement in the nutritional status and quality of life of children with CP, the present study aimed to relate the nutritional status and the variables associated to the feeding of children and adolescents diagnosed with spastic quadriplegic CP treated at the Outpatient Clinic for Special Patients at Hospital de Clínicas de Uberlândia, Universidade Federal de Uberlândia (HC-UFU), Uberlândia City, Minas Gerais State. More specifically, the objective was to check the total energy value ingested and the route of administration of the diet; describe the frequency of complications associated to eating; assess which are the complications in the administration of oral and enteral diets; and analyze the relation between complications and nutritional status.

## METHOD

This is a cross-sectional study, carried out with 28 children and adolescents, aged ≤13 years old, diagnosed with spastic quadriplegic CP and cared for between July 5^th^, 2016 and January 17^th^, 2017 by the nutrition team of the Outpatient Clinic for Special Patients at HC-UFU - an institution that serves roughly 45 children with this diagnosis per year. All patients included in the study had the consent form signed by their legal guardian, and there was no withdrawal from participation in research.

For data collection, the analysis of medical records was performed and an instrument developed by the researchers was used, which included a general form, containing information about the patient (patient code, gender, date of birth, age, time of follow-up at the clinic, date of inclusion in research and clinical diagnosis described in medical records); nutritional assessment form (weight, height, knee height, estimated height, body mass index - BMI, classification on growth curves, energy needs and protein needs); and food assessment form (feeding route, 24-hour recall, total caloric value of the 24-hour recall and complications related to food described in medical records).

For anthropometric measurement, weight was measured, and height was estimated for later BMI. Weight was obtained by subtracting the child’s weight from the parent responsible for the parent’s weight when alone. For children and adolescents who did not present spinal deformations, the measurement of height was performed according to the method of Lohman et al.;^9^ for those who did not walk, did not stand or had postural problems, height was estimated using the equation proposed by Kuperminc et al.,^10^ with the measurement of knee height.

The classification of nutritional status was performed according to the specific growth curves for children and adolescents with CP.^11^ Individuals who were below the 10^th^ percentile were classified as underweight for age, small for age or malnourishment; those who were between the 10^th^ and 90^th^ percentiles, were considered with appropriate weight for their age, appropriate height for age or eutrophic; and those who were above the 90^th^ percentile, with high weight for age, big for age or overweight - according to the weight/age, height/age and BMI/age curves, respectively.

In the present study, the calculations of the basal metabolic rate (BMR), the total energy requirement and the protein requirement for each patient were performed, according to what was proposed by the World Health Organization (WHO),^12^ and data on the patient’s feeding route and the 24-hour recall was applied to analyze food intake, total caloric value calculation and protein intake. In addition, the guardians were asked to report information about the route of administration, the type of diet administered, if any and what were the complications related to the administration of the diet and/or during feeding. Such data were written down on specific forms.

The diets adopted by the patients were classified as homemade, mixed or industrialized. Children on homemade diets received only the food used by their family, either orally or enterally, and did not receive any type of industrialized formula. Children on mixed diets received their family’s common food plus the industrialized formula appropriate to the age of each child. Those who received industrialized diets had the formula as exclusive food. For the analysis of the adequacy of energy and protein intake, Acceptable Macronutrient Distribution Values (AMDR) were used. AMDR are conceptualized by the acceptable distribution of dietary macronutrients in percentages associated to reduced risk of chronic diseases; they take into account the life cycle in which the individual is inserted and are based on the appropriate relation between macronutrients and energy to maintain an adequate energy balance. The energy adequacy range considers values between 60 and 100% of the total caloric value, and the protein range, between 10 and 30% of it. If an individual consumes more than what is recommended by the AMDR, there is a potential increase in the risk of the occurrence of chronic diseases. On the other hand, if they consume less than it, they may be deficient in essential nutrients. Thus, the values stipulated by the AMDR limit the maximum and minimum intake of macronutrients.^13^

Statistical analyzes were performed using the SISVAR software for Windows (Universidade Federal de Lavras, Lavras City, Brazil), Statistical System R (University of Auckland, New Zealand) and Statistical Package for the Social Sciences (SPSS) for Windows, version 16.0 (University of Chicago, Chicago, United States). For descriptive analysis of data, the average was calculated. First, the proportions were analyzed to check if they followed a normal distribution with the Shapiro-Wilk test.^14^ For cases in which data normality was not observed, that is, in which there were asymmetric distributions, the median and the confidence interval for median were estimated. The correlations between the variables analyzed were estimated by Spearman’s correlation with their respective p-values. Those with p <0.05 were adopted as significant correlations.

The paper was approved by the Research Ethics Committee of UFU under protocol number 046455/201, and it follows the principles of the Declaration of Helsinki for research involving human beings.

## RESULTS

The participants’ mean age was 7.0 years (95% CI 5.75-8.32). A total of 25% of participants (n=7) fit in group 4 of CP (do not walk, do not speak and eat orally), and 75% (n=21), in group 5 (do no walk, do not speak and eat alternatively) (Table 1).

**Table 1:** Characteristics of children and adolescents with spastic quadriplegic cerebral palsy treated at the Outpatient Clinic for Special Patients at Hospital de Clínicas de Uberlândia, Universidade Federal de Uberlândia.

Variable	N	%
Gender		
Male	14	50
CP Group		
Group 4*	7	25
Group 5^†^	21	75
Cause of CP		
Neonatal asphyxia	6	21
*Kernicterus*	4	14
Congenital diseases	3	11
CNS Malformation	3	11
Other causes	5	18
No report	7	25
Nutritional status		
Eutrophy	16	57
Malnutrition	11	39
Overweight	1	4
Feeding route		
Gastrostomy	19	68
OR	7	25
NEP	2	7

CP: cerebral palsy; CNS: central nervous system; OR: oral route; NEP: nasoenteral probe; *group 4: patients who do not walk, do not speak, and eat orally; ^†^group 5: patients who do not walk, do not speak, and do not eat orally; ^§^sepsis, neonatal cytomegalovirus, head trauma, hypoxic encephalopathy.

Among the causes of CP, neonatal asphyxia, congenital diseases, such as toxoplasmosis and rubella; malformation of the central nervous system (CNS); kernicterus; sepsis; neonatal cytomegalovirus; head trauma; and hypoxic encephalopathy were reported (Table 1). Table 2 also describes the characterization of the nutritional status and the diet of the study participants.

**Table 2:** Associations between nutritional diagnosis and the type of diet used by children and adolescents with spastic quadriplegic cerebral palsy treated at the Outpatient Clinic for Special Patients of Hospital de Clínicas de Uberlândia, Universidade Federal de Uberlândia.

Type of diet	Nutritional status		
	Malnutrition (%) CI	Eutrophy (%) CI	Overweight (%) CI
Homemade	50 [6.8-93.2]	25 [0.6-80.6]	25 [0.6-80.6]
Industrialized	60 [14.7-94.7]	40 [5.3-85.3]	0 [0.0-52.2]
Mixed	33.3 [13.3-59.0]	66.7 [41.0-86.7]	0 [0.0-18.5]

CI: confidence interval.

The most frequent complications observed were oropharyngeal dysphagia (39%, n=11), intestinal constipation (39%, n=11) and GERD (39%, n=11). Furthermore, the same patient could have had two or more complications. The presence of recurrent diarrhea was less frequent among the patients evaluated (4%, n=1) (Table 3).

**Table 3:** Frequency of food-related complications in children and adolescents with spastic quadriplegic cerebral palsy treated at the Outpatient Clinic for Special Patients at Hospital de Clínicas de Uberlândia, Universidade Federal de Uberlândia.

Complication	n	%
Oropharyngeal dysphagia	11	39
GERD	11	39
Intestinal constipation	11	39
Recurrent vomiting	9	32
Recurrent flu	5	18
Aspiration pneumonia	4	14
Recurrent infections	2	7
Recurrent diarrhea	1	4

GERD: gastroesophageal reflux disease.

When assessing nutritional status according to BMI, 4% (n=1) of the children were overweight; 39% (n=11) were malnourished, and 57% (n=16) were classified as eutrophic. On the weight-for-age curve, 11% (n=3) of the children were overweight; 25% (n=7), underweight, and 64% (n=18) of children had age-appropriate weight. Regarding height, 14% (n=4) were tall for their age, and 86% (n=24) presented height appropriate for their age. As to the frequency of complications associated to nutritional status and the diet, no significant association was observed (Table 4).

**Table 4:** Correlation between the frequency of complications related to feeding with the nutritional status and with the feeding route of children and adolescents with spastic quadriplegic cerebral palsy treated at the Outpatient Clinic for Special Patients of Hospital de Clínicas de Uberlândia, Universidade Federal de Uberlândia.

Complication	Nutritional status r (p-value)	Feeding route r (p-value)
Oropharyngeal dysphagia	0.20 (0.310)	-0.15 (0.432)
GERD	-0.07 (0.737)	-0.15 (0.432)
Intestinal constipation	0.07 (0.737)	0.28 (0.155)
Recurrent vomiting	-0.35 (0.070)	0.35 (0.066)
Recurrent flu	0.25 (0.192)	-0.19 (0.333)
Aspiration pneumonia	0.08 (0.665)	0.15 (0.434)
Recurrent infections	0.00 (1.000)	-0.17 (0.394)
Recurrent diarrhea	0.17 (0.374)	-0.30 (0.114)

r: Spearman’s correlation; GERD: gastroesophageal reflux disease. Spearman’s correlation was used, with p <0.05 as significant.

As for the feeding route, 25% of children with CP ate orally, and 75% used alternative route (nasoenteral tube or gastrostomy). Regarding the analysis of the frequency of diets used by patients with CP, 14.2% (n=4) were identified with a homemade diet, 17.8% (n=5), with an industrialized diet, and 57.1% (n=19) with a mixed diet (homemade and industrialized) (Table 4).

The correlation between caloric adequacy, macronutrient content and the volume of the diet offered to patients was statistically significant and positive between the diet received and the nutritional status of patients (0.48; p=0.01). On the other hand, there was a negative correlation between the diet versus the nutritional status of patients (-0.17; p=0.4), making it possible to infer that the diet used did not influence the nutritional status of patients.

When assessing energy needs compared to dietary records, 50% (n=14) of individuals ingested energy amounts above what is considered ideal, and 14.2% (n=4), inferior amounts than the calculated energy requirement. When assessing the distribution of energy adequacy in relation to nutritional status, 28.6% (CI 13.2-48.7) of the eutrophic patients and 3.6% (CI 1.0-18.3) of the malnourished ones had an adequate energy intake (p=0.009). As for protein intake, most (68%) of the individuals evaluated had an adequate intake following the recommendations described by age, according to the AMDR.^13^

## DISCUSSION

Among individuals with CP and the classification of the characteristics of functionality and of the feeding route, there is a uniform distribution between groups 4 and 5 (groups that present greater impairment of their functions). The prevalence of individuals in group 5 was 35.6%, and in group 4, 34.6%.^15^ However, these data differed from those found in the present study, which showed a higher proportion of individuals in group 5 (75% in group 5 versus 25% in group 4), suggesting that the pattern of patients seen at the HC-UFU Outpatient Clinic for Special Patients was of more committed, more serious individuals, and that, in most cases, require specialized interdisciplinary follow-up due to the need to use an alternative feeding route.

In the present study, the main cause of CP was neonatal asphyxia (21%, n=6), an event that could be minimized with good prenatal care and during delivery.^16^ Volpe et al.,^16^ in a study on the causes of CP, show a similar result, with asphyxia being responsible for the largest contingent of cerebral impairment in newborns, in addition to being the first cause of neonatal neurological morbidity. After asphyxia, kernicterus is the second most prevalent cause of CP in our population (14%, n=4), followed by congenital diseases (11%, n=3) and CNS malformation (11%, n=3). Just like what was observed in the study by Rotta et al.,^17^ the most frequent congenital infections detected in this study were rubella and toxoplasmosis.

We also observed that most individuals evaluated were classified as eutrophic (57%), followed by malnourished (39%), with emphasis on the use of growth curves specific to the health condition of the patients analyzed. Literature documents properly that nutritional status has a significant impact on the health and quality of life of individuals with CP, leading to a greater need for health care.^18^ It is usual for pediatric patients with CP to be classified according to their nutritional status in non-specific growth curves. Nonetheless, this practice should be reviewed due to the possibility of using specific curves for these patients, which consider delayed motor development and the use of the feeding route. Thus, individuals with eutrophic CP may have been considered malnourished because of the use of routine curves for unaffected children.

Even when using specific curves, the prevalence of malnutrition is frequent. In addition, worsening in nutritional status is observed over the years and with the degree of impairment caused by CP.^19^ Dahl et al.^19^ conducted a study with 35 children and adolescents with CP and indicated a prevalence of 46% of malnourished people. The present study, on the other hand, showed that 39% of the patients analyzed were malnourished. Despite the small difference (39% versus 46%), the higher prevalence of eutrophic patients suggests that interdisciplinary work, performed in the outpatient clinic of the present study, can favor the nutritional status of patients.

From the anthropometric and dietary data obtained by the 24-hour recall, it was possible to relate the type of diet received and the route of administration of the diet with the nutritional status of patients with CP. The present study verified that the type of diet offered to children with CP positively influenced the nutritional status of these patients. It was observed that, of the four children who received the homemade diet, only one had an inadequate nutritional status (overweight). This fact can be explained by the lack of standardization observed in the preparation of diets by parents/caregivers in the ingredients used and in the homemade measures,^20^ often causing nutritionally unbalanced diets (rich in calories and low in nutrients). Another important aspect possibly related to overweight in children is the little physical activity identified for this group of patients, which generates low energy expenditure.

Out of those children diagnosed with malnutrition, 60% received only industrialized diets. Such a result was not expected by the researchers, since the industrialized enteral diet suitable for the age is easier to be prepared and, therefore, there are less chances of having great differences between what was prescribed and what was offered. Among the possible reasons for this finding, the incorrect preparation by parents/caregivers is highlighted, who may offer amounts below the prescribed, or error during the dilution process. Grammatikopoulou et al.^21^ compared children with CP with their siblings with normal development and showed that the caloric intake of children with CP was insufficient, which could be explained by the difficulty and the time spent by caregivers to offer meals. In addition, another group of researchers found that the greater the degree of impairment of the child, the lower the energy intake, which can lead to malnutrition.^22^ Thus, the values of malnutrition found by the researchers were also evidenced in similar studies and in groups of patients with the same characteristics.

For children who received mixed diets, most of them were eutrophic. This result can be justified by the greater easiness for parents preparing children’s meals, because they could use the common family preparations in some meals and the formula in others. Another important factor that may have contributed to the eutrophic state of children who received mixed diets was the constant monitoring of children with CP in the specialized outpatient clinic. The adaptations made by the multiprofessional team at each consultation, and whenever necessary, contributed to the maintenance of an adequate nutritional status.

It is of utmost importance that during the bibliographic survey carried out by the researchers to support the discussion, no research relating the nutritional status of patients with CP, the types of diet and the feeding routes was identified. Considering the relevance of the topic and the results found by the researchers, further research should be developed to better assist patients with CP.

In the present study, no statistically significant correlation was identified between the nutritional status of patients and the route used for the administration of diets, which could be explained by the reduced number of the study sample. It is well described in the literature that home enteral nutrition (via alternative feeding) is the best option for children with some type of disability, when nutritional therapy is needed for long periods.^23^ Strauss et al. identified that the use of gastrostomy increased children’s life expectancy in seven years.^24^ A review carried out in 2015 by Nelson et al.^25^ found that there are positive effects (weight gain and greater comfort for the child) and negative effects (the use of alternative route requires great care on the part of caregivers) in relation to the use of gastrostomy.^25^ Usually, children who use only the oral route to eat have greater difficulty in gaining weight. It has also been observed that feeding exclusively by oral route is stressful for parents/caregivers, because each meal takes, on average, three hours to be carried out and, often, food is wasted.^21^

The nutritional status of patients with CP is affected by complications related to food, and the use of alternative routes is a strategy to be adopted.^26^ In a study by Marchand et al.^27^ with neurologically compromised patients who were unable to feed themselves, it was demonstrated that they consumed less than the recommended amount of energy. Although most patients evaluated had an alternative feeding route, there was no statistical difference from those who were on oral feeding as to the frequency of these complications, suggesting effectiveness in the nutritional guidelines performed by the team.

During the nutritional consultation, the parents or guardians are advised about the food that should be administered to patients, as well as the time, the way of preparation, the manner in which this administration should be carried out and what should be done in case of any complication.

Some of the evaluated participants (33%, n=9) consumed less protein than that recommended for their age, according to the AMDR.^13^ Similar results were demonstrated by Lopes et al.,^28^ who found that 46% of children and adolescents with spastic CP consumed low protein amounts. Inverse results were observed by Sangermano et al.,^29^ who found that protein intake was above the recommended level. However, it is important to highlight that these latter authors^29^ used, as an adequacy range, values between 12 and 15% of the total caloric value of the diet, and not the one proposed by the AMDR (10 to 30%).^13^

As for complications related to food, the most frequent among children and adolescents with CP were oropharyngeal dysphagia, GERD and intestinal constipation, similar to those found by Sullivan et al.^30^ Such complications, when severe, can negatively impact food intake and favor nutritional deficit.^30^ The prevalence of oropharyngeal dysphagia among children and adolescents with CP varies between authors. In a study by Parker et al.^31^ in 1,357 children and adolescents with CP, the presence of oropharyngeal dysphagia was observed in 19.9% of them. Another study, carried out by Penagini et al.^18^ with individuals with quadriplegic CP, indicated dysphagia in 12.8% of the individuals. On the other hand, in the paper by Calis et al.^32^ with 166 participants with greater motor impairment, 99% of those affected with oropharyngeal dysphagia were observed. The results observed in the present study, with severely compromised patients, showed a frequency of 39% (n=11). These values are at odds with Calis et al.^32^ However, this difference may be related to the sample size (n=28), and further studies with more participants may be needed to confirm such data.

Oropharyngeal dysphagia in CP is associated to worse nutritional conditions, resulting in less food intake and, consequently, higher occurrence of malnutrition. In addition, oropharyngeal dysphagia in CP favors the occurrence of aspiration pneumonia and other recurrent respiratory infections. Thus, it becomes the first indication for an alternative feeding route, in order to improve the nutritional and health prognosis of these individuals.^18^ In this study, 39% (n=11) of patients had GERD. Literature data indicate that the prevalence of this disease can reach up to 75%.^33^ The diagnosis of GERD in patients with CP is important, because it can lead to a reduction in caloric intake due to the presence of recurrent vomiting and food refusal. In addition to nutritional damage, GERD can result in esophageal inflammation, dental erosions and increased risk of aspiration, which can worsen the patient’s nutritional status.^30^ ^,^ ^34^

Intestinal constipation is one of the three main complications that affect individuals with CP, aggravated by prolonged immobility, postural deformities and the stiffness of the abdominal muscles, which hinder peristalsis, and low intake of fluids and fibers.^30^ ^,^ ^35^ The prevalence of intestinal constipation observed in the evaluated patients (39%) is in accordance with the literature (26-74%).^20^ ^,^ ^25^ The use of the alternative route (tube or gastrostomy) can facilitate the administration of fluids and diet, improving constipation.

Although the association of dietary complications with nutritional status and diet has not been demonstrated, knowledge of the complications related to diet in pediatric and adolescent patients with CP is important and may allow the creation of strategies to prevent nutritional impacts. One of them is the use of alternative feeding routes, but this can be ineffective without support and adequate nutritional guidelines.

Among the limitations of the present study, the non-questioning regarding the use of multivitamins and/or medications that can influence the individual’s nutritional status is highlighted; and the method of calculating energy and protein needs applied the criteria established by the WHO. Such criteria are established for healthy children, which may underestimate or overestimate energy needs, because there may be patients with high caloric expenditure due to spasticity or low caloric expenditure due to inactivity. However, although there are some specific formulas for calculating BMR validated in scientific studies, the real energy and protein needs for children with CP are not completely elucidated in the scientific literature. Notwithstanding, there was a small number of individuals assessed and with subjectivity in the report of the 24-hour recall, which made its calculation difficult. Therefore, two soup recipes were developed and calculated to be used in the guidelines for outpatient patients, in order to standardize the guidelines and facilitate preparation and calculations.

The nutritional status of children and adolescents with CP is affected by several factors inherent to their own condition, and the complications associated to eating have a negative impact on the lives of these patients, requiring not only alternative feeding routes, but also continuous interdisciplinary work to promote health and quality of life. In addition, the results obtained in the present study reinforce the need for continuous nutritional guidance for the parents/caregivers of the children in the study, as well as the choice of an adequate feeding route for each child/adolescent by the multiprofessional team that accompanies them.
